# Living-Donor Liver Transplant and Improved Post-Transplant Survival in Patients with Primary Sclerosing Cholangitis

**DOI:** 10.3390/jcm12082807

**Published:** 2023-04-11

**Authors:** Leandro Sierra, Romelia Barba, Bryan Ferrigno, Daniela Goyes, Wilfor Diaz, Vilas R. Patwardhan, Behnam Saberi, Alan Bonder

**Affiliations:** 1 Division of Gastroenterology, Hepatology, and Nutrition, Beth Israel Deaconess Medical Center, Boston, MA 02215, USA; lsierrac@bidmc.harvard.edu (L.S.); rbarbabe@bidmc.harvard.edu (R.B.); vpatward@bidmc.harvard.edu (V.R.P.); bsaberi@bidmc.harvard.edu (B.S.); 2 Department of Medicine, Beth Israel Deaconess Medical Center, Boston, MA 02215, USA; bwferrig@bidmc.harvard.edu (B.F.); wdiazfer@bidmc.harvard.edu (W.D.); 3 Department of Medicine, Loyola Medicine—MacNeal Hospital, Berwyn, IL 60402, USA; daniela.goyesvaca@luhs.org

**Keywords:** primary sclerosing cholangitis, autoimmune liver diseases, liver transplant, graft survival

## Abstract

Primary sclerosing cholangitis (PSC) is the leading indication of liver transplantation (LT) among autoimmune liver disease patients. There is a scarcity of studies comparing survival outcomes between living-donor liver transplants (LDLT)s and deceased-donor liver transplants (DDLTs) in this population. Using the United Network for Organ Sharing database, we compared 4679 DDLTs and 805 LDLTs. Our outcome of interest was post-LT patient survival and post-LT graft survival. A stepwise multivariate analysis was performed, adjusting for recipient age, gender, diabetes mellitus, ascites, hepatic encephalopathy, cholangiocarcinoma, hepatocellular carcinoma, race, and the model for end-stage liver disease (MELD) score; donor’ age and sex were also included to the analysis. According to univariate and multivariate analysis, LDLT had a patient and graft survival benefit compared to DDLT (HR, 0.77, 95% CI 0.65–0.92; *p* < 0.002). LDLT patient survival (95.2%, 92.6%, 90.1%, and 81.9%) and graft survival (94.1%, 91.1%, 88.5%, and 80.5%) at 1, 3, 5, and 10 years were significantly better than DDLT patient survival (93.2%, 87.6%, 83.3%, and 72.7%) and graft survival (92.1%, 86.5%, 82.1%, and 70.9%) (*p* < 0.001) in the same interval. Variables including donor and recipient age, male recipient gender, MELD score, diabetes mellitus, hepatocellular carcinoma, and cholangiocarcinoma were associated with mortality and graft failure in PSC patients. Interestingly, Asians were more protected than Whites (HR, 0.61; 95% CI, 0.35–0.99; *p* < 0.047), and cholangiocarcinoma was associated with the highest hazard of mortality (HR, 2.07; 95% CI, 1.71–2.50; *p* < 0.001) in multivariate analysis. LDLT in PSC patients were associated with greater post-transplant patient and graft survival compared to DDLT patients.

## 1. Introduction

Primary sclerosing cholangitis (PSC) is an immune-mediated chronic cholestatic liver disease with an incidence of 1.11 per 100,000 persons in the US population and 0.77 per 100,000 persons globally [1]. PSC primarily affects intra- and extrahepatic bile ducts, causing progressive inflammation and obliterative fibrosis, resulting in multiple stricture formations [2]. It has a highly variable clinical presentation, and over half of PSC patients are asymptomatic at diagnosis. Aggressive disease is characterized by recurrent episodes of biliary tract obstruction and cholangitis that may ultimately progress to cirrhosis, liver failure, or hepatobiliary malignancies [3].

Currently, no immunosuppressive or disease-modifying agents are available to prevent PSC patients from progressing to end-stage liver disease (ESLD) [4]. Liver transplantation (LT) is the only proven treatment to prolong survival among PSC patients with ESLD [5]. According to the United Network for Organ Sharing (UNOS) database, from 2008 to 2016, PSC patients accounted for 48% of the total liver transplants among autoimmune liver disease (ALD) patients, which has been increasing over the last several years. Among ALDs, PSC is now the leading indication for LT [6].

Living-donor liver transplantation (LDLT) is a potential alternative option to bridge the current organ supply–demand mismatch. While it has been performed for the last 20 years, LDLT accounts for only a small minority (3–4%) of adult LTs in the US [7]. The rates of LDLTs are higher among patients with PSC compared to LT among other forms of ESLD [8]. Previously, research surrounding the benefit of LDLT has largely focused on the reduction in waitlist mortality for LDLT recipients who avoid prolonged waitlist times; it is unknown whether there are potential long-term benefits of receiving a living- versus deceased-donor graft. Outcomes research regarding survival differences among types of LT for patients with PSC are scarce and raise the importance of long-term analyses [9,10,11]. With this study, we aimed to compare survival rates between LDLT and deceased-donor liver transplants (DDLTs) among patients with PSC.

## 2. Materials and Methods

### 2.1. Study Population

We conducted a comprehensive retrospective cohort study using the UNOS Organ Procurement and Transplantation Network (OPTN) database to identify adult patients with PSC who received liver transplants between 27 February 2002, and 31 December 2019. To ensure the accuracy and specificity of our findings, we excluded pediatric patients (aged below 18 years), individuals with a history of previous transplants, those who received multiorgan transplants, and patients with associated autoimmune liver diseases. The data reported here were supplied by the UNOS as the contractor for the OPTN. The interpretation and reporting of these data are the responsibility of the author(s) and in no way should be seen as an official policy of or interpretation by the OPTN or the US government. Given that UNOS is a publicly available deidentified patient-level database, institutional review board approval was not required according to the policies of the UNOS and Beth Israel Deaconess Medical Center.

### 2.2. Outcomes

The primary focus of our study was to analyze the survival of patients after liver transplantation. This outcome was defined as the length of time between the date of transplant and the death of the recipient. Additionally, we assessed post-transplant graft survival as our secondary outcome, which was defined as the length of time between the transplant date and either graft failure or the need for a re-LT.

### 2.3. Study Variables

Patient characteristics were compared between DDLT and LDLT groups. Recipient and donor characteristics differed and were analyzed separately. A larger number of variables was considered for the recipient subcohort, including age at the time of liver transplantation, gender, self-reported race/ethnicity, body mass index (BMI), blood type, MELD score at the time of transplantation, history of diabetes mellitus (DM), hepatocellular carcinoma (HCC), cholangiocarcinoma (CCA), hepatic encephalopathy (HE), ascites, and UNOS regions. Within the donor variables, we considered the age at the time of transplant and gender. PSC was extracted using the codes in the Scientific Registry of Transplant Recipient (SRTR) dictionary.

### 2.4. Statistical Analysis

We stratified clinical and demographic characteristics by donor type and presented categorical variables using numbers and percentages. Continuous variables were expressed as mean and standard deviation (SD). We utilized chi-square testing to evaluate associations among categorical variables and T-tests to compare continuous variables. To analyze survival outcomes, we employed Kaplan–Meier methods.

To identify significant predictors of survival, we conducted forward stepwise multivariate Cox regression analyses, which were adjusted for both recipient and donor characteristics. We included variables that were statistically significant at the bivariate level (partial regression (0.1) and partial elimination (0.05)) or were known to be clinically relevant, such as recipient age at the time of transplantation, gender, race, diabetes mellitus, and the model for end-stage liver disease (MELD) score at the time of transplantation. Additionally, we considered the donor’s age as a factor in our analysis. The results are presented as hazard ratios (HRs) with 95% confidence intervals (CIs), and statistical significance was defined as α = 0.05. All statistical analyses were conducted using Stata version 17.0 (StataCorp LP, College Station, TX, USA).

## 3. Results

Our study identified a total of 5484 patients diagnosed with PSC who received an LT between 2002 and 2019. The cohort characteristics are presented in Table 1. Notably, 14% of patients with PSC received LDLT. The majority of the population was Caucasian males. DDLT recipients were found to be significantly older than LDLT recipients (48 (13.5) vs. 44 (14); *p* < 0.001). Furthermore, the mean MELD score was significantly higher in the DDLT group than in the LDLT group (22 vs. 15; *p* < 0.001). According to the data, the regions with the highest rates of PSC among DDLT recipients were 3, 10, and 11, while the regions with the highest rates among LDLT recipients were 2, 5, and 7. However, it is important to note that these findings only provide the number and proportion of PSC cases for each group and do not offer an explanation for why these rates differ across regions based on demographics. Our analysis also revealed that DDLT recipients were more likely to have hepatic encephalopathy and ascites than LDLT recipients. Notably, the presence of cholangiocarcinoma was significantly lower in patients in the DDLT group compared to the LDLT group (5% vs. 7%; *p* = 0.03).

**Figure 1:**
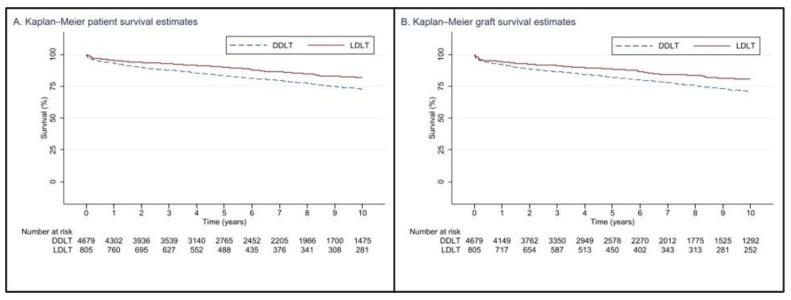
Kaplan–Meier curves demonstrating post-transplant patient and graft survival when analyzed by donor type.

The patient survival rates for LDLT in this study at 1, 3, 5, and 10 years (95.2%, 92.6%, 90.1%, and 81.9%, respectively; *p* < 0.001) were significantly better than those for DDLT recipients (93.2%, 87.6%, 83.3%, and 72.7%, respectively; *p* < 0.001) (Figure 1). Similarly, there was a graft survival benefit at 1, 3, 5, and 10 years for LDLT (94.1%, 91.1%, 88.5%, and 80.5%, respectively; *p* < 0.001) compared to DDLT recipients (92.1%, 86.5%, 82.1%, and 70.9%, respectively; *p* < 0.001).

We conducted univariate Cox regression analysis to examine post-transplant patient and graft survival, the results of which are presented in Table 2. Our findings demonstrate that LDLT recipients were associated with improved survival relative to DDLT recipients (HR, 0.66; 95% CI, 0.56–0.79; *p* < 0.001). However, certain characteristics, such as older recipient age (HR, 1.09; 95% CI, 1.05–1.12; *p* < 0.001), older donor age (HR, 1.22; 95% CI, 1.17–1.27; *p* < 0.001), and male recipient gender (HR, 1.14; 95% CI, 1.02–1.28; *p* = 0.025), were associated with decreased patient survival. Additionally, diabetes mellitus (HR, 1.61; 95% CI, 1.39–1.87; *p* < 0.001), ascites (HR, 1.16; 95% CI, 1.03–1.29; *p* = 0.01), hepatic encephalopathy (HR, 1.19; 95% CI, 1.07–1.32; *p* < 0.001), hepatocellular carcinoma (HR, 1.43; 95% CI, 1.15–1.77; *p* < 0.001), and cholangiocarcinoma (HR, 2.07; 95% CI, 1.71–2.50; *p* < 0.001) were all associated with post-transplant mortality. Furthermore, we found that Hispanic patients had a higher risk of mortality compared to White patients (HR, 1.29; 95% CI, 1.00–1.66; *p* = 0.046).

Moreover, other results suggest that LDLT is associated with better post-transplant graft survival compared to DDLT (HR, 0.67; 95% CI, 0.57–0.79; *p* < 0.001). Patient characteristics including recipient age (HR, 1.18; 95% CI, 1.14–1.24; *p* < 0.001), donor age (HR, 1.10; 95% CI, 1.07–1.14; *p* < 0.001), and male recipient gender (HR, 1.16; 95% CI, 1.03–1.30; *p* = 0.012) were associated with worse graft survival outcomes. Variables such as diabetes mellitus (HR, 1.56; 95% CI, 1.35–1.81; *p* < 0.001), ascites (HR, 1.14; 95% CI, 1.02–1.27; *p* = 0.025), hepatic encephalopathy (HR, 1.19; 95% CI, 1.07–1.32; *p* < 0.001), hepatocellular carcinoma (HR, 1.36; 95% CI, 1.09–1.68; *p* = 0.006), and cholangiocarcinoma (HR, 2.05; 95% CI, 1.70–2.47; *p* < 0.001) had deleterious effects on post-transplant graft survival. Interestingly, Asians had a post-transplant graft survival advantage (HR, 0.59; 95% CI, 0.35–0.99; *p* = 0.05).

In stepwise multivariate analyses, the recipient’s age (HR, 1.18; 95% CI, 1.13–1.23; *p* < 0.001) and donor´s age (HR, 1.06; 95% CI, 1.03–1.09; *p* < 0.001) were found to have adverse effects on patient survival (refer to Table 3). Additional variables linked to a detrimental impact on patient survival were diabetes mellitus (HR, 1.41; 95% CI, 1.21–1.64; *p* < 0.001), hepatocellular carcinoma (HR, 1.35; 95% CI, 1.09–1.69; *p* = 0.007), and cholangiocarcinoma (HR, 2.22; 95% CI, 1.84–2.69; *p* < 0.001). Furthermore, compared to Whites, Hispanics had a higher mortality risk (HR, 0.59; 95% CI, 0.35–0.99; *p* < 0.047), while Asians had a post-transplant graft survival advantage (HR, 0.59; 95% CI, 0.35–0.99; *p* < 0.047).

A stepwise multivariate analysis was also conducted to examine post-transplant graft survival (Table 4). Results showed that LDLT recipients had better post-transplant graft survival outcomes than those with DDLT (HR, 0.75; 95% CI, 0.63–0.90; *p* = 0.002). In addition, the age of the recipient (HR, 1.13; 95% CI, 1.08–1.18; *p* < 0.001), the age of the donor (HR, 1.07; 95% CI, 1.04–1.08, *p* < 0.001), and male gender of the recipient (HR, 1.12; 95% CI, 1.00–1.26; *p* < 0.045) had deleterious effects on graft survival. Additionally, diabetes mellitus (HR, 1.39; 95% CI, 1.20–1.62; *p* < 0.001), hepatic encephalopathy (HR, 1.12; 95% CI, 1.00–1.25; *p* < 0.041), hepatocellular carcinoma (HR, 1.31; 95% CI, 1.05–1.63; *p* = 0.016), and cholangiocarcinoma (HR, 2.19; 95% CI, 1.81–2.65; *p* < 0.001) were found to have a detrimental effect on graft survival. In particular, cholangiocarcinoma was associated with the highest hazard of mortality in PSC transplant recipients.

## 4. Discussion

A critical aspect of treating PSC is liver transplantation, as it is the only definitive therapy available. However, LT is a limited resource, with nearly 20% of waitlisted patients either dying or being removed due to clinical deterioration each year [12,13]. Despite this, there is a lack of studies comparing outcomes between DDLT and LDLT, specifically in the PSC patient population. One multicenter study conducted over 15 years found no significant difference in patient and graft survival between DDLT and LDLT for patients with PBC [14]. A smaller, single-centered study also found similar results, with no significant difference in survival between the two transplant types [15]. Additionally, a previous UNOS database analysis for the period of 2002 to 2006 revealed that after adjustment for MELD score and recipient age, there was no statistically significant difference in survival between the two types of transplants for PSC patients; however, unadjusted survival was superior in the LDLT group [16]. It should be noted that UNOS facilitated the identification of the population diversity by dividing the country into 11 different regions. Each region is confirmed by various states, and their purpose is to provide an effective mechanism of governance and operational effectiveness within the OPTN. To the best of our knowledge, this is the first study using the UNOS database displaying trends toward improved post-transplant outcomes in patients with PSC who underwent DDLT versus LDLT after adjustment for multiple variables.

Our study demonstrates a significant patient and graft survival benefit favoring LDLT compared to DDLT in PSC. Patients who underwent LDLT had a 25% reduced risk of post-transplant mortality and graft failure after conducting a stepwise multivariate analysis than those who underwent DDLT. Factors that were independently associated with post-transplant patient and graft survival were the donor and recipient ages at the time of transplantation, the recipient’s sex, Asian race, MELD score, diabetes, HCC, and CCA before transplantation. This is in contrast with a cohort study conducted by Heinemann et al. in which the European Liver Transplant Registry was used. In this analysis, LDLT patients had an increased risk of death from graft failure compared to DDLT patients after adjusting for recipient age, sex, and the era of LT. Interestingly, in this study, other autoimmune liver diseases were also analyzed; however, associations between adverse graft outcomes and LT type were only seen in PSC recipients [17].

A few aspects are important to consider with respect to the recipient characteristics. Asian race is a protective survival factor, with a post-transplant patient mortality risk and graft survival risk of 42% less than all other races. Kemmer et al. strongly supported the findings of our findings in a study using a 2002–2010 interval of the UNOS database [18]. In the mentioned analysis, Asian patients had the highest 5-year graft and patient survival rates compared to other races; however, this was evaluated in liver transplant recipients of all liver diseases and not specifically in PSC patients [19]. Unlike the significant survival benefit in Asians, self-reported Hispanic patients had worse survival outcomes, with a higher mortality risk compared to other races. In contrast to our study, a recent analysis by Thuluvath et al. showed that the Hispanic race had better graft and patient survival when compared to Caucasians, with hazard ratios of 0.88 and 0.90, respectively [19]. It is important to note that this study considered all adults available with an LT, sufficient data, and no presence of re-LT [19].

Estimated patient survival and graft survival before and after adjusting for the mentioned variables appear to be superior in those who underwent LDLT compared to DDLT. It is interesting to highlight that although higher MELD scores significantly increase the mortality risk, the impact is relatively minimal and does not seem to have clinical relevance. Moreover, while previous studies using the UNOS database have shown differences in waitlist time between LDLT and DDLT groups [20,21,22], our present study found no difference in waitlist time between LDLT and DDLT for PSC patients, despite the significant difference in MELD score between the groups. This may be due to the relatively low symptom burden in patients with PSC [20], which makes them less likely to experience clinical deterioration or be removed from the waitlist [23].

There are several hypotheses for our findings regarding the potential patient and graft survival benefits of LDLT. One possibility is that the surgical centers performing LDLT are typically higher-volume centers with well-connected resources. Thus, the surgical and subsequent graft outcomes at these centers compared to those that only perform DDLT may be superior. Second, while data were mixed, large database studies have shown that older donor age at the time of liver transplantation is a risk factor for graft failure, which appears to be the case for PSC patients based on our analysis [16,24]. Increasing donor age when analyzing all liver transplants (including DDLT and LDLT) has been shown to place recipients at an increased risk for both biliary complications [24]. Furthermore, as mentioned, emerging evidence suggests that increasing donor age also carries an increased risk of vascular complications and potentially worse mortality compared to younger donors [24,25].

Several theories have been proposed to explain the superior graft survival observed in LDLT patients compared to DDLT patients. One important factor to consider is the Donor Risk Index (DRI), which can vary significantly between LDLT and DDLT donors and may contribute to differences in graft failure risk [7,26]. Another key consideration is the potential for selection bias among experienced transplant teams, who may be more likely to recommend LDLT or select certain recipients based on various factors [26,27]. While these factors may primarily benefit graft survival in LDLT patients, they can also confer advantages for patient survival in this population [26].

The strengths of our study include its analysis of a large-scale database of transplant candidates over an extended period. Additionally, to the best of our knowledge, few studies have looked specifically at post-transplant patients and graft survival in patients with PSC. There are some limitations to our study. First, there are significant differences in the baseline characteristics between the live-donor and deceased-donor groups. While our model was adjusted for many variables, given that PSC is a progressive disease, there may be some multicollinearity between age and MELD scores, which would lessen the precision of our findings. Second, it should be considered the retrospective nature of our study and the lack of granularity inherent found in this large, nationwide database. Thirdly, the limitation in the data that were available for review creates difficulties in drawing more conclusive arguments.

## 5. Conclusions

Our study provides compelling evidence that LDLT offers superior patient and graft survival outcomes in patients with PSC compared to DDLT. Specifically, our findings suggest that LDLT is associated with lower mortality risk after adjusting for multiple variables. However, we also found that recipient age, male sex, MELD score, and certain comorbidities can negatively impact LDLT patient survival, while older donors can be associated with higher mortality. Further investigation is needed to explore the effects of other variables on long-term patient and graft survival.

## Figures and Tables

**Table 1:** Cohort characteristics.

	DDLT	LDLT	*p*-Value
Recipient Characteristics			
Age, ±mean (SD)	48.4 (13.5)	43.9 (14)	<0.001
Gender, male n (%)	3182 (68)	514 (64)	0.020
Race/ethnicity, n (%)			<0.001
Caucasian	3598 (77)	716 (89)	
Black/African American	756 (16)	47 (5)	
Hispanic	188 (4)	34 (4)	
Asian	94 (2)	5 (0.6)	
Other	43 (0.9)	3 (0.4)	
BMI, ±mean (SD)	26 (5)	25 (4)	<0.001
Blood type, n (%)			<0.001
0	2047 (44)	389 (48)	
A	1743 (37)	342 (42)	
B	672 (14)	68 (8)	
AB	217 (5)	6 (1)	
MELD score, ±mean (SD)	22.3 (9.1)	15 (5.7)	<0.001
Diabetes mellitus, n (%)	516 (11)	70 (9)	0.048
Hepatocellular carcinoma, n (%)	262 (6)	22 (3)	0.001
Cholangiocarcinoma, n (%)	233 (5)	55 (7)	0.030
Hepatic Encephalopathy, n (%)	2259 (48)	290 (36)	<0.001
Ascites, n (%)	3107 (66)	402 (49)	<0.001
Waitlist time, ±mean (SD)	394 (672.65)	371 (662.18)	0.358
Region, n (%)			<0.001
1	110 (2)	80 (10)	
2	424 (9)	128 (16)	
3	832 (18)	9 (1)	
4	337 (7)	38 (5)	
5	459 (10)	116 (14)	
6	189 (4)	0 (0)	
7	267 (6)	74 (9)	
8	442 (9)	67 (8)	
9	267 (6)	74 (9)	
10	660 (14)	53 (7)	
11	505 (11)	29 (4)	
Donor characteristics			
Age, ±mean (SD)	41 (17.3)	38 (10.3)	<0.001
Gender, male n (%)	2743 (59)	403 (50)	<0.001

DDLT—deceased-donor liver transplant; LDLT—living-donor liver transplant; SD—standard deviation; MELD—model for end-stage liver disease.

**Table 2:** Univariate Cox proportional hazard analyses of predictors of post-transplant patient survival and post-transplant graft survival.

Post-Transplant Patient Survival				Post-Transplant Graft Survival		
Variable	Hazard Ratio	95% CI	*p*-Value	Hazard Ratio	95% CI	*p*-Value
Donor type						
DDLT	[Reference]			[Reference]		
LDLT	0.66	0.56–0.79	0.000	0.67	0.57–0.79	0.000
Recipient age	1.09	1.05–1.12	0.000	1.18	1.14–1.24	0.000
Male gender	1.14	1.02–1.28	0.025	1.16	1.03–1.30	0.012
Race						
White	[Reference]			[Reference]		
Black/AA	1.04	0.89–1.21	0.609	1.03	0.89–1.20	0.674
Hispanic	1.29	1.00–1.66	0.046	1.28	0.99–1.65	0.054
Asian	0.61	0.36–1.03	0.062	0.59	0.46–1.00	0.052
Other	0.91	0.47–1.76	0.789	0.90	0.46–1.72	0.741
Diabetes mellitus	1.61	1.39–1.87	0.000	1.56	1.35–1.81	0.000
MELD	1.01	1.00–1.02	0.000	1.01	1.00–1.02	0.001
Ascites	1.16	1.03–1.29	0.011	1.14	1.02–1.27	0.025
Hepatic encephalopathy	1.19	1.07–1.32	0.001	1.19	1.07–1.32	0.001
Cholangiocarcinoma	2.07	1.71–2.50	0.000	2.05	1.70–2.47	0.000
Hepatocellular carcinoma	1.43	1.15–1.77	0.001	1.36	1.09–1.68	0.006
Donor age	1.22	1.17–1.27	0.000	1.10	1.07–1.14	0.000
Male donor gender	0.95	0.86–1.06	0.378	0.94	0.86–1.03	0.192

CI—confidence interval; DDLT—deceased-donor liver transplant; LDLT—living-donor liver transplant; MELD— model for end-stage liver disease.

**Table 3:** Stepwise multivariate Cox proportional hazard analyses of predictors of post-transplant patient survival.

Variable	Hazard Ratio	95% CI	*p*-Value
Donor type			
DDLT	[Reference]		
LDLT	0.75	0.63–0.90	0.002
Recipient age	1.18	1.13–1.23	0.000
Race			
White	[Reference]		
Hispanic	1.29	1.00–1.66	0.047
Asian	0.58	0.34–0.99	0.045
Diabetes mellitus	1.41	1.21–1.64	0.000
MELD	1.01	1.00–1.02	0.000
Cholangiocarcinoma	2.22	1.84–2.69	0.000
Hepatocellular carcinoma	1.35	1.09–1.69	0.007
Donor age	1.06	1.03–1.09	0.000

CI—confidence interval; DDLT—deceased-donor liver transplant; LDLT—living-donor liver transplant; MELD—model for end-stage liver disease.

**Table 4:** Stepwise multivariate Cox proportional hazard analyses of predictors of post-transplant graft survival.

Variable	Hazard Ratio	95% CI	*p*-Value
Donor type			
DDLT	[Reference]		
LDLT	0.75	0.63–0.90	0.002
Recipient age	1.13	1.08–1.18	0.000
Male sex	1.12	1.00–1.26	0.045
Race			
White	[Reference]		
Asian	0.58	0.34–0.98	0.042
Diabetes mellitus	1.39	1.20–1.62	0.000
MELD	1.01	1.00–1.01	0.014
Hepatic encephalopathy	1.12	1.00–1.25	0.041
Cholangiocarcinoma	2.19	1.81–2.65	0.000
Hepatocellular carcinoma	1.31	1.05–1.63	0.016
Donor age	1.07	1.04–1.08	0.000

CI—confidence interval; DDLT—deceased-donor liver transplant; LDLT—living-donor liver transplant; MELD—model for end-stage liver disease.

## Data Availability

Data can be requested at https://unos.org/data/ (accessed on 17 January 2023).
